# Investigation of Changes in Tooth Colour After the Use of Different Aligners

**DOI:** 10.7759/cureus.71047

**Published:** 2024-10-07

**Authors:** Abdullah Arslan, Muzaffer Gülyurt

**Affiliations:** 1 Orthodontics, Biruni University, Istanbul, TUR; 2 Orthodontics and Dentofacial Orthopaedics, Biruni University, Istanbul, TUR

**Keywords:** aligner, attachment, discolouration, fluid composite, stability

## Abstract

Clear aligner treatment is an orthodontic treatment that is custom-made with 3D printers without the use of conventional metal or porcelain wires and brackets. It is a modern orthodontic method used to correct tooth positions that need to be corrected aesthetically and functionally.

Aim

This study aimed to compare the discolouration of teeth and clear aligners after 15 days of full-time use of clear aligners.

Materials and methods

The 20 patients in the study group had used clear aligners for 45 days, including three different clear aligners for 15 days. In addition, 20 patients were included as a control group to measure the difference in tooth discolouration.

Retaining attachments using G-Aenial (GC Corp, Tokyo, Japan) and 3M ESPE (St. Paul, MN, USA) flowable composites were placed crosswise to each patient in the distal regions of all lateral and canine teeth. The performance of three aligner plates (Zendura Flex (Bay Materials LLC, Fremont, CA, USA); Duran (ScheuDental GmbH, Iserlohn, Germany); and Taglus (Laxmi Dental Export Pvt. Ltd, Mumbai, India)), two fluid composite attachments, and two clear aligner cleaners regarding discoloration were tested. Each aligner was used for 15 days, and then the patient was moved to the next aligner. Colour change on the aligners and the patients' teeth were assessed from the canine to the middle third and mid-plane of the canine tooth using a VITA Easyshade V spectrophotometer (Bad Säckingen, Germany). An aligner cleaner was used immediately after the patients used their clear aligners, and then the measurements of the aligner color values were completed. The 120 aligners obtained were divided into two groups. Sixty aligners were treated with Fresh Guard (Efferdent, Lynchburg, VA, USA), and the other 60 were treated with Corega (GlaxoSmithKline, London, UK) tablets and stored in a glass of water.

Results

The colour stability of G-Aenial Flo was higher than that of the 3M ESPE fluid composite (p=0.040 and p=0.024). Our study determined that staining occurred in all the teeth we measured as a result of using aligner. Fresh Guard aligner cleaner caused more discoloration on Zendura and Duran aligners than Corega aligner cleaner (p<0.05). Taglus plaque was found to have more discoloration than Zendura and Duran aligner (p<0.05).

Conclusion

In conclusion, Duran clear aligners are preferable to other clear aligners in terms of discoloration. In addition, G-Aenial Flo can be recommended as an attachment or fluid composite because of its better color stability results.

## Introduction

Numerous previous studies have investigated the mechanical and aesthetic properties of biomaterials for their application in the orthodontic field [1]. Nowadays, thermoplastic materials are widely used in the production of clear aligners due to their excellent properties [2]. In particular, polyester, copolyester, polycarbonate, thermoplastic polyurethanes and polypropylene are the predominant thermoplastic material compositions used for the production of clear aligners [3]. These materials allow the fabrication of highly accurate devices through a thermoforming process on accurate models of patient malocclusions. However, studies on aligners in a simulated intraoral environment and on aligner samples taken after intraoral exposure have indicated that these devices do not retain their original shape or composition in the mouth.

From an aesthetic point of view, the colour stability and transparency of orthodontic clear aligners should be stable over two-week orthodontic treatment periods [4]. However, the colour stability of dental materials is often affected by various factors such as ultraviolet irradiation, coloured drinks and mouthwashes [5]. During use, it is recommended to remove the aligners before eating and drinking. However, studies have shown that patient compliance with removable orthodontic appliances is insufficient, and this is often a matter of concern for orthodontists [6].

Exposure of the tray to staining agents in the oral cavity is inevitable for users who drink without removing the tray from their mouth, especially due to time constraints during work. In patients who do not follow the instructions, pigments in the staining agents can accumulate and cause discolouration of the aligner materials. Thus, clear aligners may become less aesthetically appealing even during two-week treatments and this is a clinical concern. Therefore, there is a need to investigate the colour stability of commonly used aligner types to provide evidence for clinical aesthetic considerations and guidelines for both patients and orthodontists [7].

The aim of this study was to compare the colour changes of clear aligners and teeth after 15 days of full-time intraoral use of clear aligners in patients.

## Materials and methods

In the study group, 20 patients used three different transparent aligners for 15 days for a total of 45 days. In addition, 20 patients were taken as a control group to measure the difference in tooth colouration.

Patients in the study group used aligner, while patients in the control group did not. The patients in the control group were not used with any attachment, and were moved from the maxillar and mandibular canine teeth to the canine teeth on the first, 16th, 31st and 46th days.

**Figure 1:**
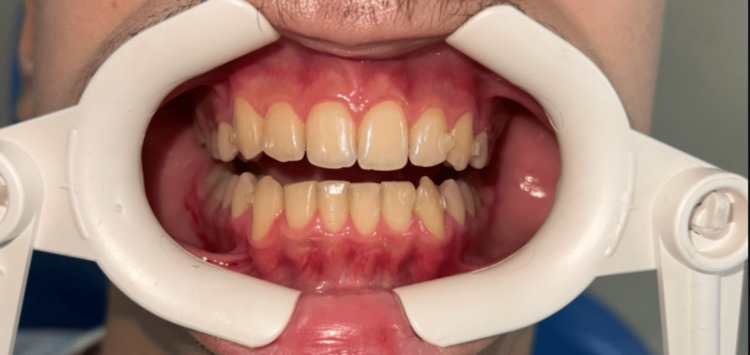
View of the inserted retainer attachments in the patient's mouth

Attachments using G-Aenial (GC Corp, Tokyo, Japan) and 3M ESPE (St. Paul, MN, USA) flowable composites were placed diagonally on the distal areas of all lateral and canine teeth in each patient (Table 1, Figure 1).

**Table 1:** Fluid composites applied for attachments and their properties

Material	Type	Content	Filler	Filler Content	Colour	Manufacturer
Filtek™ Bulk Fill	Light-cure bulk-fluid	BisGMA, UDMA, BisEMA, prokrilat resins	Zirkonya/silika, Yterbium Triflouride	64,5/42	A2	3M ESPE, St.Paul, MN USA
G-Aenial Flo	Universal Fluid	UDMA, Bis-MEPP, TEGDMA,	Silikon Dioxide, Stroniium glass	69/50	A2	GC Corp., Tokyo, Japan

Filtek Bulk Fill fluid composite was used for the first (upper right canine and upper right lateral) and third (lower left canine and lower left lateral) attachments, while G-Aenail fluid composite was used for the second (upper left canine and upper left lateral) and fourth (lower right and canine and lower right lateral) attachments (Table 2).

**Table 2:** Table of fluid composites in empty regions without attachments and in regions with attachments

13	12	11	21	22	23
3M ESPE	3M ESPE	None	None	GC	GC
GC	GC	None	None	3M ESPE	3M ESPE
43	42	41	31	32	33

Colour measurements were made and saved before the application in the first session. Oral hygiene training was given in the same session.

After the attachment was placed on the teeth, transparent aligners were prepared on the intraoral models obtained from the measurements taken from the patient's mouth. Zendura Flex (Bay Materials LLC, Fremont, CA, USA) was used for the first clear aligner, Duran (ScheuDental GmbH, Iserlohn, Germany) was used as the second aligner material and Taglus (Laxmi Dental Export Pvt. Ltd, Mumbai, India) was used as the third aligner material. Each aligner was used for 15 days and then another aligner was used.

**Figure 2:**
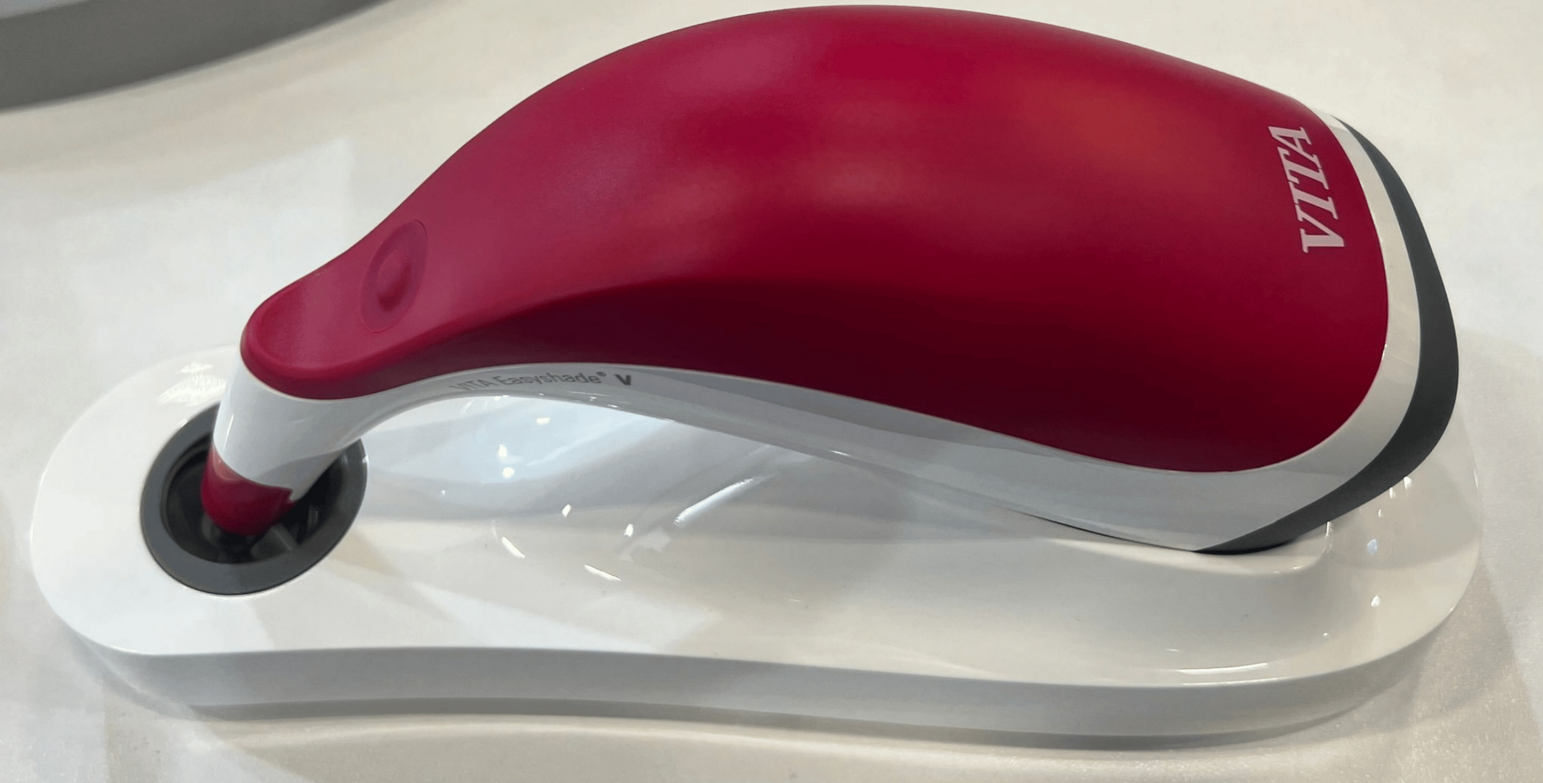
Vita Easyshade V Spectrophotometer used for colour measurement in the study

On the first, 16th, 31st and 46th day of the application, measurements were made by holding the device at a 90-degree angle at the midpoint of the upper and lower central, lateral and canine teeth of the patients. VITA Easyshade V (VITA Classical; VITA Zahnfabrik, Bad Sackingen, Germany) digital spectrophotometer was used for all measurements (Figure 2).

**Figure 3:**
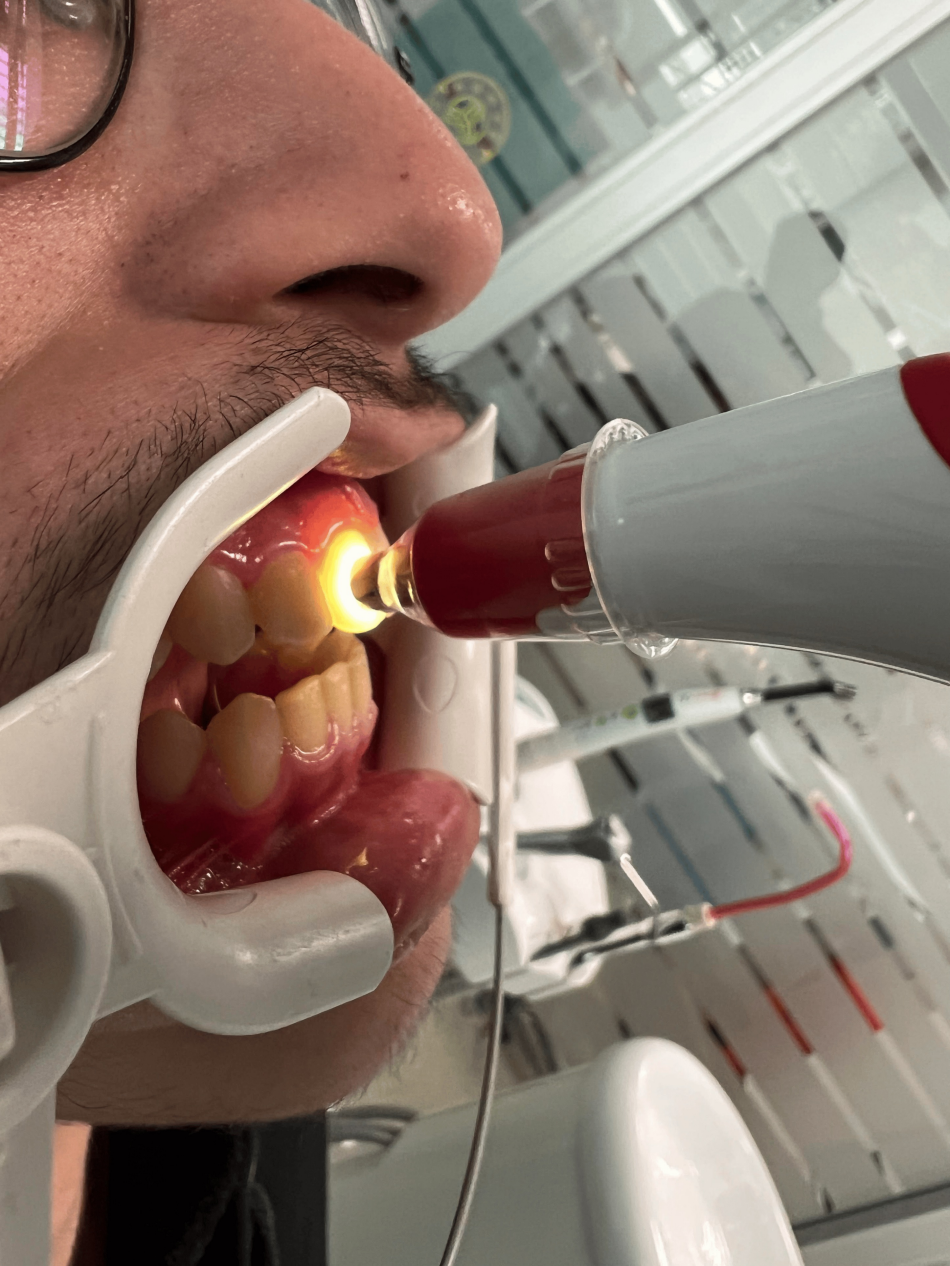
VITA Easyshade V spectrophotometer measurement of a patient in the control group

No tooth movement was performed during the application. At the end of the application, the attachments were removed from the teeth and the particles were cleaned. For accuracy assessment, three measurements were taken from the same area, arithmetic mean values were taken and it was ensured that the spectrophotometer charge was not less than 75% during the measurement. An orthodontic retractor was used for all measurements made on the patient in order to avoid any fluid, saliva, etc. (Figure 3).

All measurements for tooth colour were performed by a single operator under standard test conditions. Before any measurement, the instrument was calibrated on its own white ceramic block. According to the International Commission on Illumination (CIE) standard, daylight illumination conditions (Biruni University Dental Hospital Clinic) were set to 6500 K and 1000 L. Natural daylight was excluded.

## Results

The distribution of colouration measurements according to composites and measurement times in the experimental group is given, Kruskal Wallis tests were used to compare the colouration measurements according to composites for times and Friedman tests were used to compare the colouration measurements according to composites for times (Table 3).

**Table 3:** Distribution and comparison of discolouration measurements for composites and measurement times in the experimental group Δ: a symbol representing a change in something

Measurement Process				
Initial measurement-After attachment	ΔL1	Δa1	Δb1	ΔE1
After attachment-Zendura	ΔL2	Δa2	Δb2	ΔE2
Zendura-Duran	ΔL3	Δa3	Δb3	ΔE3
Duran-Taglus	ΔL4	Δa4	Δb4	ΔE4
Taglus-Attachment removal	ΔL5	Δa5	Δb5	ΔE5

As a result of the analyses performed for the first, second, third and fourth measurement times, statistically significant differences were obtained between ΔL measurements according to composites (p<0.05) (Table 4).

**Table 4:** Statistical data of colouration measurements according to measurement times in the control A,B,C,a,b,c: Lilliefors significance correction *: Lower bound of the true significance Δ: a symbol representing a change in something

	ΔL1	ΔL2	ΔL3	ΔL4	ΔL5		
Composite	Mean.±S.D.(M.)	Mean.±S.D.(M.)	Mean ±S.D.(M.)	Meant.±S.D.(M.)	Mean.±S.D.(M.)	Test Stats	p
3M ESPE	2.73±3.11(2.30)^ a,A,C^	2.51±3.78(2.40)^ a,b,A,C^	2.67±3.88(3.05)^ a,b,A,B^	5.72±5.90(5.55)^ a,B^	1.67±3.55(1.25)^ a,C^	33.091	<0.001*
G-Aenial	1.19±2.60(1.30)^ b,A,C^	2.04±2.69(2.25)^ a,A,B^	2.07±3.47(1.75)^ a,A,B^	3.77±5.75(2.40)^ b,B^	0.48±3.14(0.45)^ a,C^	29.407	<0.001*
None	1.57±2.19(1.50)^ a,b,A^	3.85±3.81(3.55)^ b,B^	4.51±4.31(3.60)^ b,B^	4.11±5.17(2.65)^ a,b,B^	1.17±2.37(1.30)^ a,A^	45.697	<0.001*
Test Stats	8.633	8.530	13.248	6.480	3.583		
p	0.013*	0.014*	0.001*	0.039*	0.167		
	Δa1	Δa2	Δa3	Δa4	Δa5		
Composite	Mean.±S.D.(M.)	Mean.±S.D.(M.)	Mean ±S.D.(M.)	Mean.±S.D.(M.)	Mean.±S.D.(M.)	Test Stats	p
3M ESPE	-0,21±0,69(-0,20) ^a,A^	-0,41±0,72(-0,40)^ a,B^	-0,45±0,66(-0,30)^ a,B,C^	-0,96±1,12(-0,80)^ a,C^	-0,38±0,55(-0,40)^ a,B^	43,213	<0,001*
G-Aenial	-0,38±0,69(-0,30)^ a,A^	-0,54±0,98(-0,40)^ a,A^	-0,75±0,86(-0,50)^ b,B^	-1,06±0,91(-1,10)^ a,B^	-0,62±0,72(-0,60)^ a,A,B^	29,610	<0,001*
None	-0,33±1,01(-0,45)^ a,A^	-0,61±0,99(-0,55)^ a,A,B^	-0,69±1,13(-0,70)^ b,A,B^	-0,85±1,07(-0,90)^ a,B^	-0,26±1,11(-0,20)^ a,A^	14,795	0,005*

	Δb1	Δb2	Δb3	Δb4	Δb5		
Composite	Mean.±S.D.(M.)	Mean.±S.D.(M.)	Mean ±S.D.(M.)	Mean ±S.D.(M.)	Mean.±S.D.(M.)	Test Stats	p
3M ESPE	-1.57±3.79(-1.25)^ a,b,A,B^	-0.88±3.25(0.00)^ a,A^	-1.92±3.34(-1.4)^ a,A,B^	-2.19±3.35(-1.35)^ a,B^	-1.24±3.34(-0.90)^ a,b,A,B^	16.055	0.003*
G-Aenial	-0.6±2.76(-0.95)^ a,A,C^	-1.24±3.32(-1.3)^ a,C,D^	-1.72±3.41(-2.2)^ a,B,D^	-2.68±2.91(-2.85)^ a,B^	-2.15±2.99(-1.80)^ a,B,D^	34.035	<0.001*
None	0.01±2.96(0.05)^ b,A^	-0.86±3.21(-0.35)^ a,B^	-1.38±3.85(-2.2)^ a,B^	-1.82±3.33(-2,00)^ a,B^	-0.95±3.69(-0.35)^ b,A,B^	20.832	<0.001*
Test Stats	8.049	1.286	0.631	2.375	7.034		
p	0.018*	0.526	0,729	0.305	0.030*		
	ΔE1	ΔE2	ΔE3	ΔE4	ΔE5		
Composite	Mean.±S.D.(M.)	Mean ±S.D.(M.)	Mean.±S.D.(M.)	Mean.±S.D.(M.)	Mean.±S.D.(M.)	Test Stats	p
3M ESPE	4.99±3.06(4.7)^ a,A^	5.03±2.65(4.90)^ a,A^	5.64±2.36(5.77)^ a,b,B^	7.94±4.73(7.61)^ a,B^	4.53±2.79(3.67)^ a,A^	34.804	<0.001*
G-Aenial	3.61±1.89(3.3)^ b,A^	4.56±2.07(4.00)^ a,A,C^	5.18±2.27(4.81)^ a,C,B^	6.88±4.15(5.63)^ a,B^	4.33±2.37(4.92)^ a,A,C^	43.049	<0.001*
None	3.81±1.55(3.8)^ a,b,A,C^	5.43±3.47(5.45)^ a,C^	6.75±3.39(6.31)^ b,B^	6.48±4.20(5.55)^ a,B^	4.27±2.09(3.70)^ a,A,C^	62.570	<0.001*
Test Stats	8.833	2.231	9.969	5.232	0.011		
p	0.012*	0.328	0.007*	0.073	0.995		

According to Bonferroni tests for time first, a statistically significant difference was obtained between G-Aenial and Filtek Bulk Fill groups (p=0.015). Filtek Bulk Fill composite measurements were higher than G-Aenial composite measurements. At the second time, Bonferroni tests showed a statistically significant difference between G-Aenial and non-composite groups (p=0.015). Non-composite measurements are higher than G-Aenial composite measurements. At the third time, Bonferroni tests showed a statistically significant difference between G-Aenial and non-composite groups (p=0.001). Non-composite measurements are higher than G-Aenial composite measurements. At the fourth time, Bonferroni tests showed a statistically significant difference between G-Aenial and 3M ESPE groups (p=0.049). Filtek Bulk Fill composite measurements are higher than G-Aenial composite measurements.

As a result of the analysis for the third measurement time, a statistically significant difference was found between Δa measurements according to composites (p<0.05). According to Bonferroni tests, statistically significant differences were found between Filtek Bulk Fill and G-Aenial and no composite groups (p=0.040 and p=0.024). Filtek Bulk Fill composite measurements are higher than G-Aenial composite and non-composite measurements.

As a result of the analyses for the first and fifth measurement times, statistically significant differences were determined between Δb measurements according to composites (p<0.05).

According to the Bonferroni tests at the first time, a statistically significant difference was observed between no composite and Filtek Bulk Fill groups (p=0.014). Non-composite measurements were higher than 3M ESPE composite measurements. At the fifth time, Bonferroni tests showed a statistically significant difference between no composite and G-Aenial groups (p=0.025). Non-composite measurements are higher than G-Aenial composite measurements.

**Figure 4:**
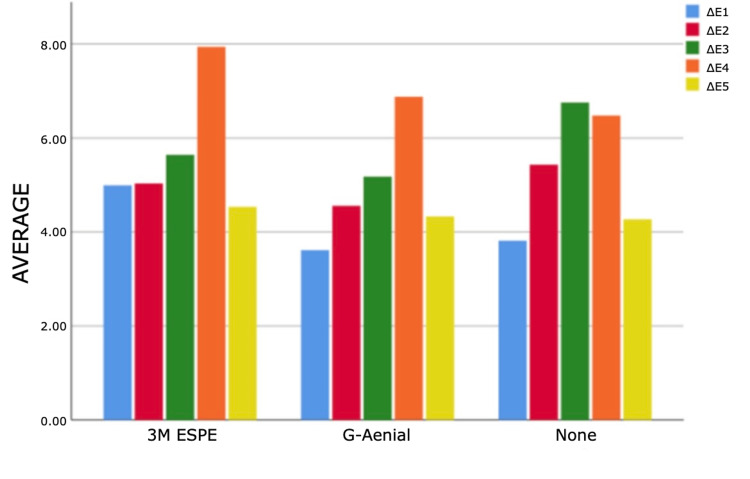
Bar graphs of the distribution of discolouration measurements according to composites and measurement times in the experimental group Δ: a symbol representing a change in something

**Figure 5:**
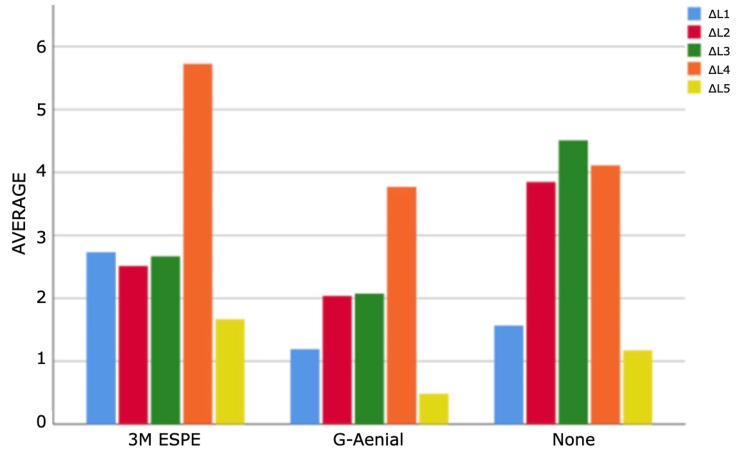
Bar graphs of the distribution of discolouration measurements according to composites and measurement times in the experimental group Δ: a symbol representing a change in something

**Figure 6:**
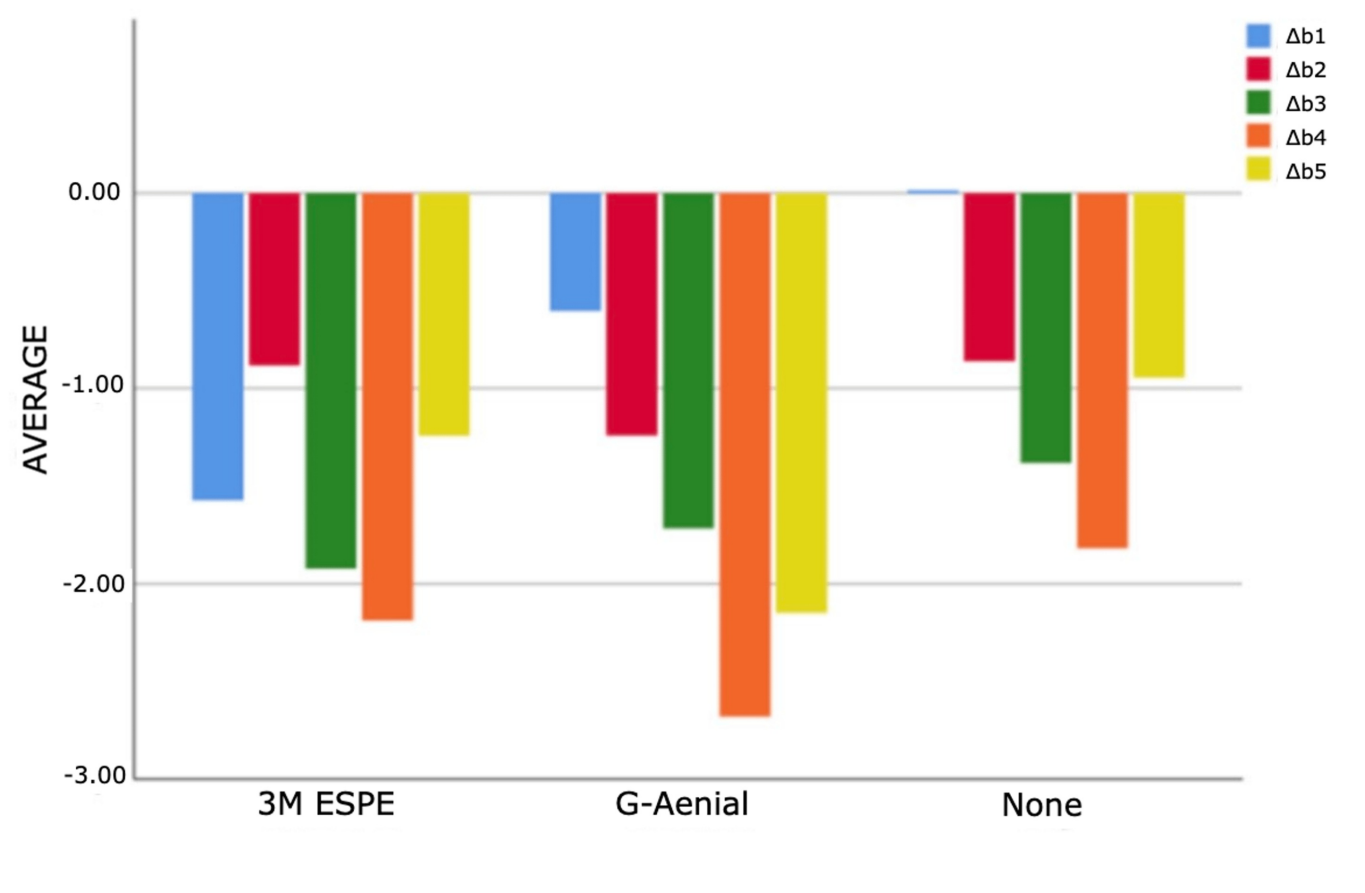
Bar graphs of the distribution of discolouration measurements according to composites and measurement times in the experimental group Δ: a symbol representing a change in something

**Figure 7:**
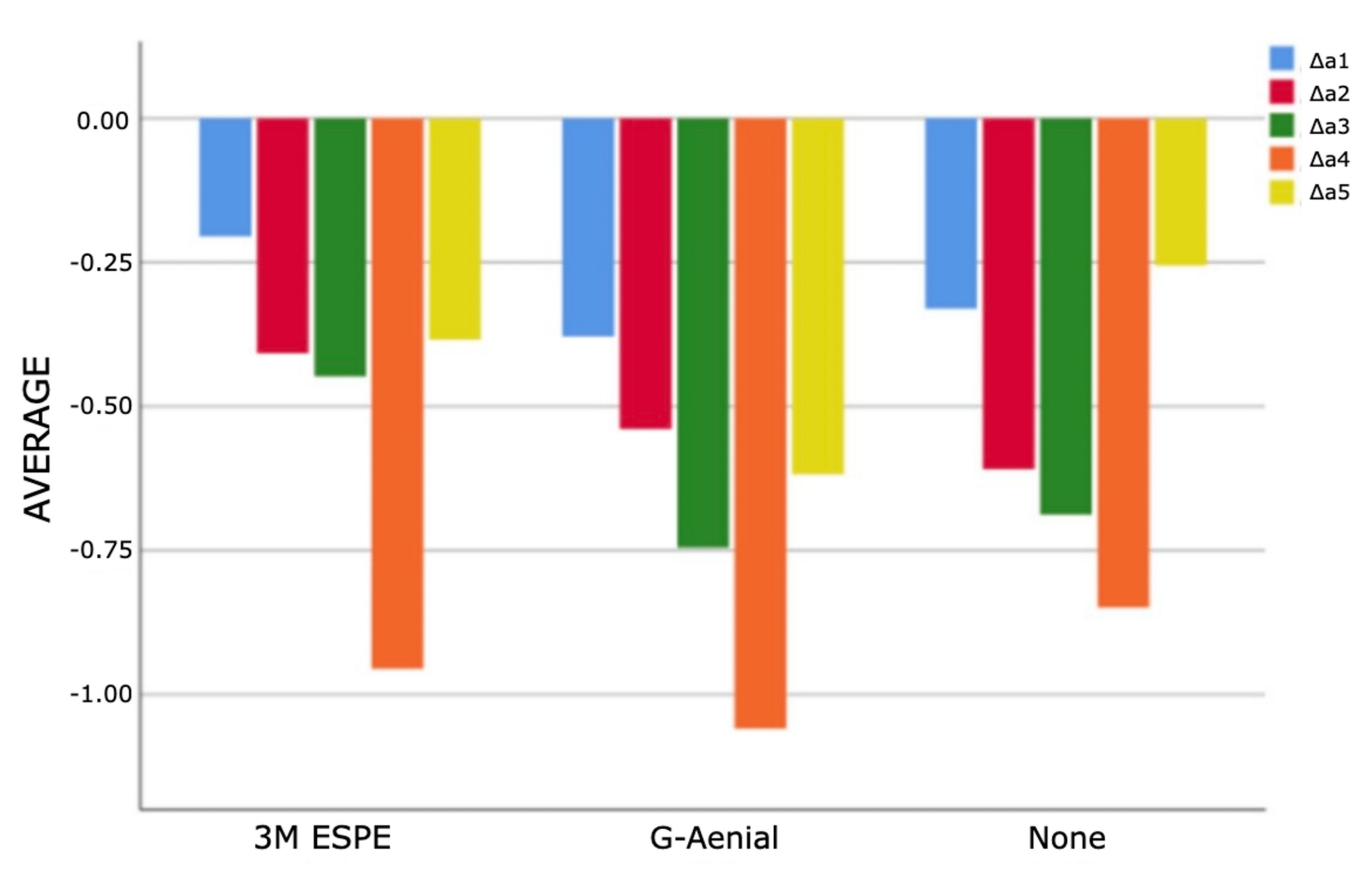
Bar graphs of the distribution of discolouration measurements according to composites and measurement times in the experimental group Δ: a symbol representing a change in something

As a result of the analyses for the first and third measurement times, statistically significant differences were found between ΔE measurements according to composites (p<0.05). According to Bonferroni tests at the first time, a statistically significant difference was found between G-Aenial and 3M ESPE groups (p=0.011). Filtek Bulk Fill composite measurements are higher than G-Aenial composite measurements. According to the Bonferroni tests for the third time, a statistically significant difference was determined between the no composite and G-Aenial groups (p=0.005). Non-composite measurements are higher than G-Aenial composite measurements (Figures 4-7).

In the control group, the distribution of colouration measurements according to the measurement times was given and Friedman tests were performed to compare the colouration measurements according to the times (Table 5).

**Table 5:** Distribution and comparison of colouration measurements according to measurement times in the control group Δ: a symbol representing a change in something

Measurement Process				
0.day-15.day	ΔL1	Δa1	Δb1	ΔE1
15.day-30.day	ΔL2	Δa2	Δb2	ΔE2
30.day-45.day	ΔL3	Δa3	Δb3	ΔE3

As a result of the analyses, a statistically significant difference was found between Δa measurements according to time (p<0.05). According to Bonferroni tests, a statistically significant difference was determined between Δa1 and Δa3 (p=0.038). Δa3 measurements are higher than Δa1 measurements (Table 6).

**Table 6:** Statistical data of colouration measurements according to measurement times in the control group A,B,C,a,b,c: Lilliefors significance correction *: Lower bound of the true significance Δ: a symbol representing a change in something

ΔL1	ΔL2	ΔL3		
Mean.±S.D.(M.)	Mean.±S.D.(M.)	Mean.±S.D.(M.)	Test stats	p
-0.01±0.33(0)	0.02±0.33(0)	0.01±0.3(0)	1.384	0.501
Δa1	Δa2	Δa3		
Mean.±S.D.(M.)	Mean.±S.D.(M.)	Mean.±S.D.(M.)	Test stats	
-0.02±0.19(0) ^a^	-0.01±0,19(0) ^a,b^	0.01±0.19(0) ^b^	9.356	0.009*
Δb1	Δb2	Δb3		
Mean.±S.D.(M.)	Mean.±S.D.(M.)	Mean.±S.D.(M.)	Test stats	p
-0.02±0.28(0)	-0.02±0.28(0)	0.02±0.32(0)	1.411	0.494
ΔE1	ΔE2	ΔE3		
Mean.±S.D.(M.)	Mean.±S.D.(M.)	Mean.±S.D.(M.)	Test stats	p
0.38±0.29(0.3)	0.37±0.29(0.3)	0.35±0.33(0.22)	2.575	0.276

**Figure 8:**
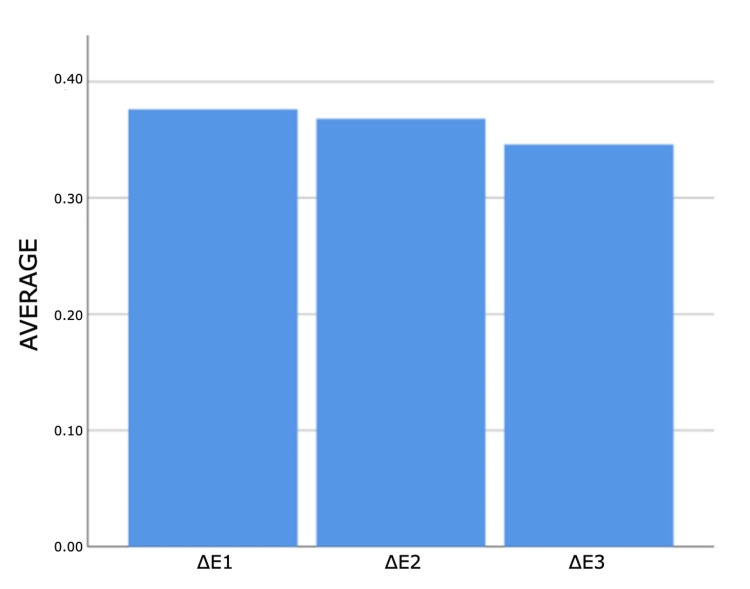
Bar graphs of the distribution of colouration measurements according to measurement times in the control group Δ: a symbol representing a change in something

**Figure 9:**
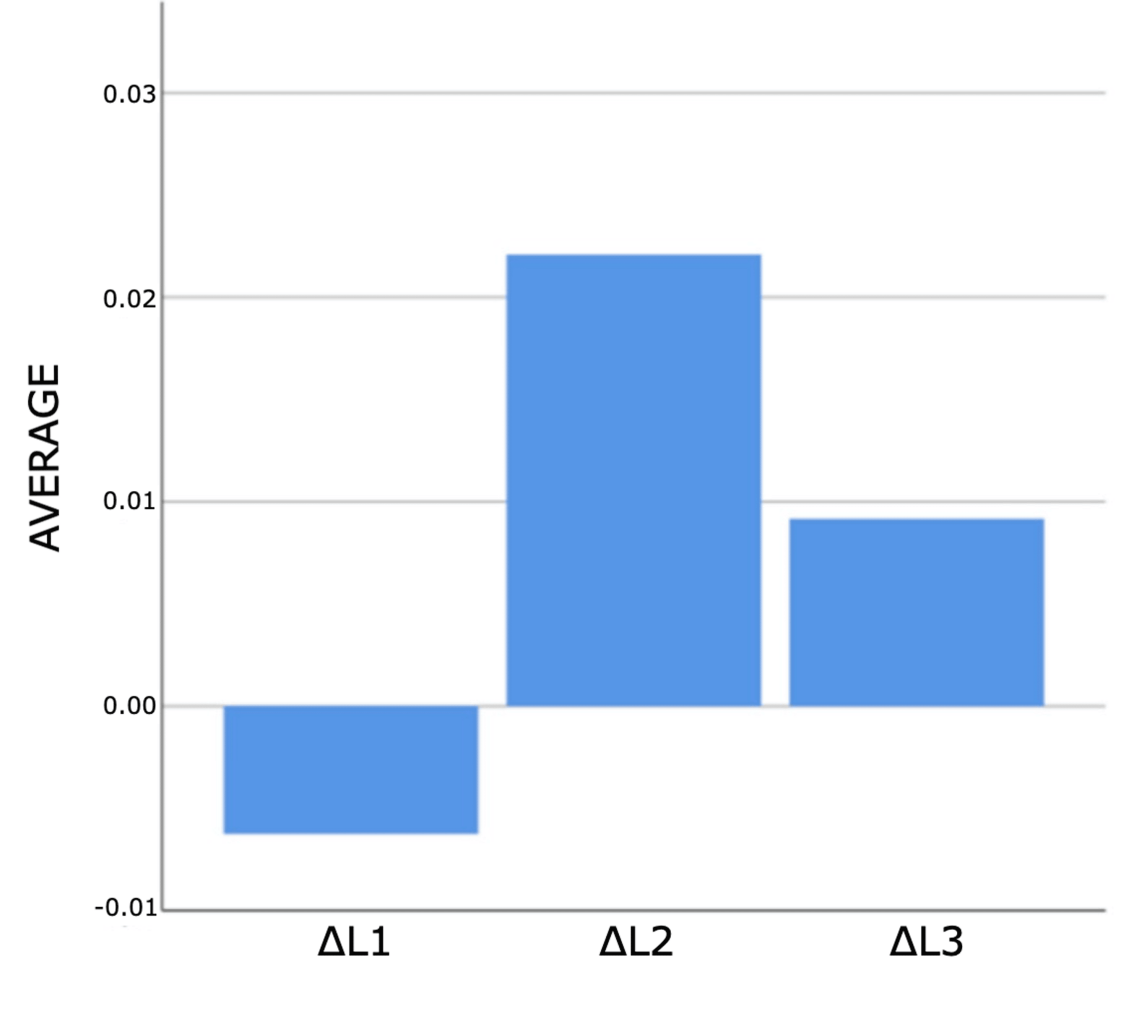
Bar graphs of the distribution of colouration measurements according to measurement times in the control group Δ: a symbol representing a change in something

**Figure 10:**
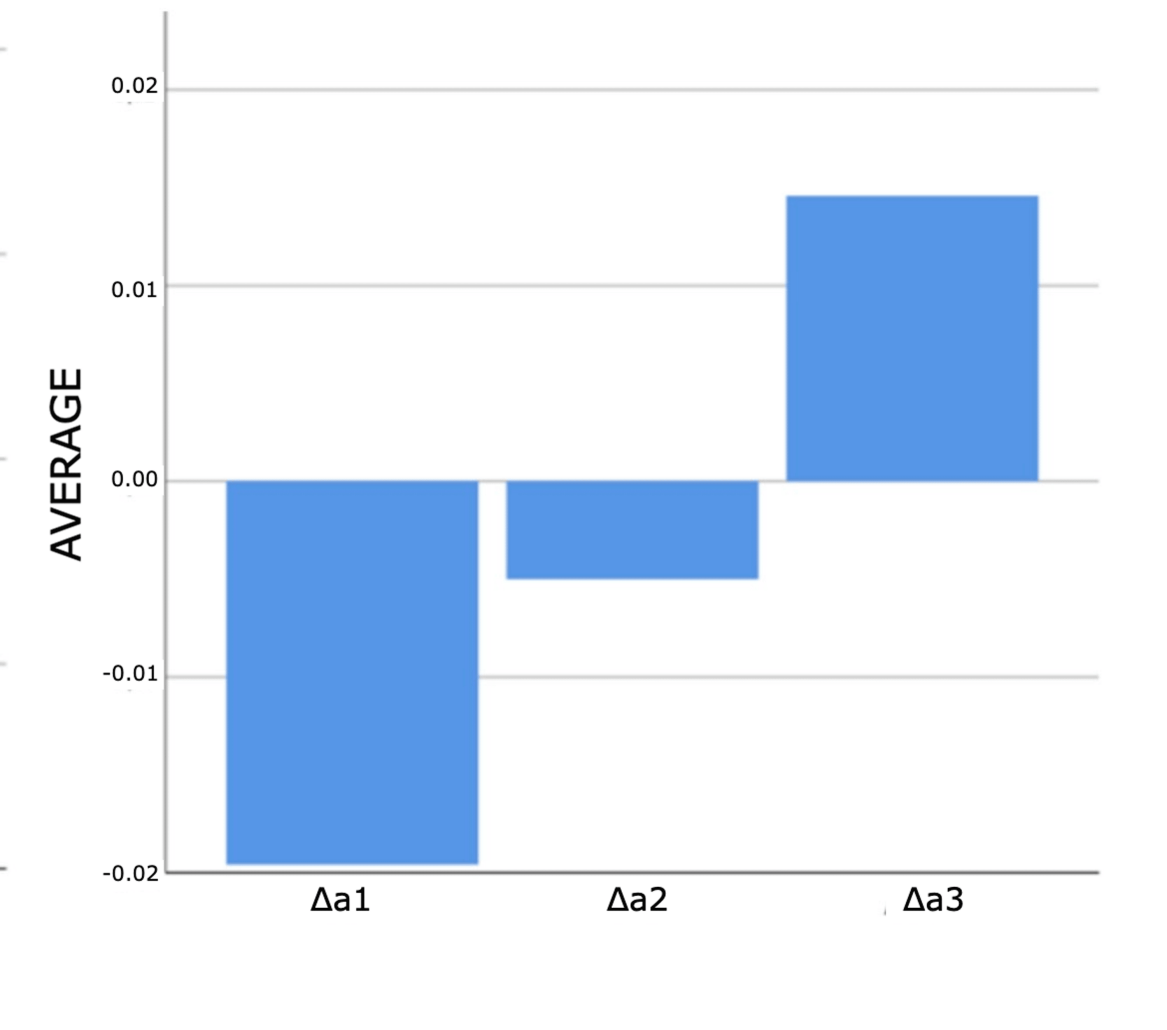
Bar graphs of the distribution of colouration measurements according to measurement times in the control group Δ: a symbol representing a change in something

**Figure 11:**
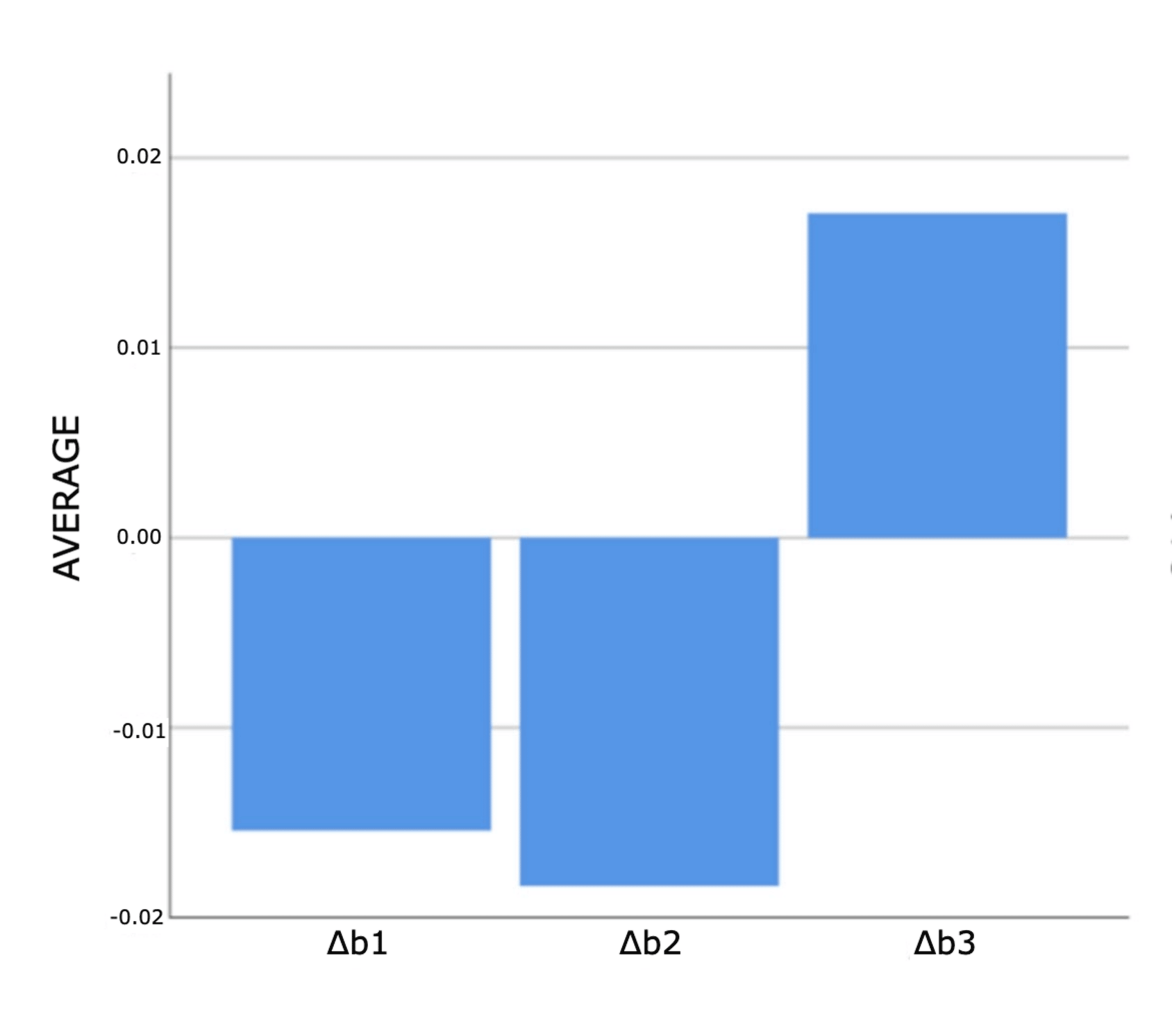
Bar graphs of the distribution of colouration measurements according to measurement times in the control group Δ: a symbol representing a change in something

No statistically significant differences were obtained between ΔL, Δb and ΔE measurements (p>0.05) (Figures 8-11).

## Discussion

With the increasing preference for aligner treatment, there is a commensurate concern about the biocompatible properties of the thermoplastic materials used. With the advancement of technology, there are several new brands of thermoplastic sheets that need to be tested. Materials such as polyurethane (PU), polyethylene terephthalate (PET) and polyethylene terephthalate glycol (PETG) are amorphous polymers that exhibit transparency properties and are commonly used in the production of transparent aligners with various applications in dentistry. PU expresses favourable properties such as ease of processing, chemical resistance and elasticity [8]. PETG is a non-crystallising amorphous copolymer of PET and is a relatively rigid material with good mechanical properties, formability, fatigue resistance and dimensional stability [9].

Since the variation of tooth colour increases from cervical to incisal and enamel-dentin tissues are of different thicknesses, the middle third of the tooth should be taken as a reference for colour selection [10].

Pusateri et al. compared the reliability and accuracy of tooth colour matching devices in a standard environment and concluded that Vita Easyshade has both reliability and accuracy values above 90% [11].

In their study, Türkün et al. used the CIELAB system for the measurement of colour difference in whitened teeth. This system is a 3D uniform colour field with equal distances corresponding to equally perceived colour differences. This system has three axes: 0 (black) to 100 (white); a* and b* represent the redness-greenness and yellowness-blueness axes respectively. When the a* and b* coordinates approach zero, the colours become neutral. Although colorimetry is another alternative mode for assessing colour change, it has the inherent disadvantage of not being able to detect colour on curved surfaces such as tooth structure. It was also found that the spectrophotometer demonstrated a high level of accuracy and repeatability. The colour change values obtained from the spectrophotometer were analysed according to CIE values. The use of L*a*b values was recommended by CIE. The reason for using these values in the study is that they are closer to human perception [12]. Measurement of colour change with a spectrophotometer and the CIE colour system allowed a quantitative and more accurate assessment than isolated visual assessment. Using a spectrophotometer in a stable environment reduces the possibility of error when recording colour. The CIELAB colour system was chosen for its ability to quantify the result [13]. A discolouration value of ∆E≥ 3.3 is defined as the threshold at which discolouration is visible to the naked eye [14]. ∆E values between 1-3.3 can be detected by some observers and ∆E values <1 are considered undetectable by a trained eye [15].

The colour temperatures of CIE simulators range from 4000K to 25000K. The most commonly used illuminant of these simulators is the D65, where D stands for daylight and 65 is the number of hundreds of the colour temperature, here 6500K. The D65 simulator with lamps is now regularly used for colour measurement [16]. In order to control both the light coming from outside and the light reflected from the background during colour measurement, colour booths with a neutral grey background and illuminated with a standard D65 daylight lamp are used.

The thickness of the aligner materials used in our study was selected as 0.76 mm. Thus, it was possible to evaluate the aligner materials in terms of colouration regardless of thickness.

The same patients used the different aligner plates used in our study. Thus, it was possible to minimise the differences in terms of blood, saliva, nutrition, drinking and stimulation.

Nanocomposites have a much larger surface area per unit mass. This can lead to staining if their interfaces are not perfectly silanised and integrated into the resin. G-Aenial Flo has ultra-fine glass fillers, which reduces the risk of filler drop-out during loading due to the small filler size and higher filler load with 69% weight compared to 20-25% in conventional flowable composites [17]. Good mechanical properties such as smooth surface finish and wear resistance were found in previous studies for G-Aenial Flo, which were attributed to reduced filler and increased resin content [18]. The production of G-Aenial Flo by a new silane process improved the hydrolytic stability and durability of the material [19]. The superior results of this flowable composite found in our study may be due to these properties of the composite.

Abzal et al. evaluated the surface roughness of three different composite resins, including the two resins in our study [20]. They found that G-Aenial Flo had the least rough surface due to the reduced filler size and uniform distribution of fillers. The flowable composite groups showed less colour change in our study. This can be attributed to the good surface property of the composite. Surface roughness can contribute to greater discolouration of composites and rougher surfaces have been found to be associated with discolouration of composite resin [21].

Discolouration can be caused by inseparable, highly cross-linkable organic networks and inorganic structures and by the ageing method that disrupts the ormocer structure [22]. G-Aenial Flo consists of a mixture of urethane dimethacrylate (UDMA) and dimethacrylate comonomers; it does not contain Bis-GMA and has been confirmed in previous studies to facilitate colour change. Bis-GMA and TEGDMA have high water absorption capacity due to their hydrophilic structure. The colour stability of UDMA has been shown to be superior to that of Bis-GMA [23]. This hypothesis supports the results of our study.

Recently, manufacturers have been producing composites with smaller filling particles in order to create materials with surface smoothness similar to tooth enamel. The lower particle size within the resin matrix produces better dispersion and smoother surfaces. Although some studies have shown that the small size of nanofilled composite resin particles allows for low staining susceptibility [24]; other researchers have reported that increasing the particle size leads to less discolouration due to a decrease in the proportion of organic filler matrix [25]. Our study supports the second hypothesis.

In the study conducted by Çörekçi et al. the individual colour variables of the teeth (L*a*b*), it was found that the L* value decreased over time, while the a* and b* values increased over time. The results of the present study similarly showed that the colour of natural teeth was affected by orthodontic treatment [26]. In our study, it was determined that L* values of natural teeth decreased over time and a* and b* values increased over time as a result of the use of aligner plates. Our study seems to support this hypothesis.

## Conclusions

Depending on the rapid advancement of science and technology in our age, many new materials and methods for treatment are developed in the field of dentistry and offered to the use of physicians. Among the treatment methods that provide advantages such as aesthetics, comfort and short treatment time, the most striking one is the treatment using aligner plates and its use is increasing rapidly day by day.

It has been proven with numerical data that the transparent aligner samples used in our study cause discolouration as a result of patient use.

Filtek Bulk Fill composite was found to cause more discolouration than G-Aenial Flo fluid composite.

This study is a unique study in terms of examining tooth discolouration in vivo after the use of aligner plates.

All these results indicate that more studies are needed in terms of in vivo human studies of aligner plates. Providing real conditions for the data obtained in this study to gain more meaning will help to understand tooth and plaque discolouration as a result of the use of aligner plates.
